# Identification of Hypoxia-Induced Genes in Human SGBS Adipocytes by Microarray Analysis

**DOI:** 10.1371/journal.pone.0026465

**Published:** 2011-10-19

**Authors:** Kathrin Geiger, Andreas Leiherer, Axel Muendlein, Nicole Stark, Simone Geller-Rhomberg, Christoph H. Saely, Martin Wabitsch, Peter Fraunberger, Heinz Drexel

**Affiliations:** 1 Vorarlberg Institute for Vascular Investigation and Treatment (VIVIT), Feldkirch, Austria; 2 Department of Medicine and Cardiology, Academic Teaching Hospital Feldkirch, Feldkirch, Austria; 3 Private University of the Principality of Liechtenstein, Triesen, Principality of Liechtenstein; 4 Division of Pediatric Endocrinology and Diabetes, University of Ulm, Ulm, Germany; 5 Medical Central Laboratories, Feldkirch, Austria; 6 Drexel University College of Medicine, Philadelphia, Pennsylvania, United States of America; Cardiff University, United Kingdom

## Abstract

Hypoxia in adipose tissue is suggested to be involved in the development of a chronic mild inflammation, which in obesity can further lead to insulin resistance. The effect of hypoxia on gene expression in adipocytes appears to play a central role in this inflammatory response observed in obesity. However, the global impact of hypoxia on transcriptional changes in human adipocytes is unclear. Therefore, we compared gene expression profiles of human Simpson-Golabi-Behmel syndrome (SGBS) adipocytes under normoxic or hypoxic conditions to detect hypoxia-responsive genes in adipocytes by using whole human genome microarrays. Microarray analysis showed more than 500 significantly differentially regulated mRNAs after incubation of the cells under low oxygen levels. To gain further insight into the biological processes, hypoxia-regulated genes after 16 hours of hypoxia were classified according to their function. We identified an enrichment of genes involved in important biological processes such as glycolysis, response to hypoxia, regulation of cellular component movement, response to nutrient levels, regulation of cell migration, and transcription regulator activity. Real-time PCR confirmed eight genes to be consistently upregulated in response to 3, 6 and 16 hours of hypoxia. For adipocytes the hypoxia-induced regulation of these genes is shown here for the first time. Moreover in six of these eight genes we identified HIF response elements in the proximal promoters, specific for the HIF transcription factor family members HIF1A and HIF2A. In the present study, we demonstrated that hypoxia has an extensive effect on gene expression of SGBS adipocytes. In addition, the identified hypoxia-regulated genes are likely involved in the regulation of obesity, the incidence of type 2 diabetes, and the metabolic syndrome.

## Introduction

White adipose tissue is a major endocrine organ and secretes various bioactive proteins – the adipokines – which are involved in different physiological processes [1]–[3]. In obesity, adipose tissue hypoxia was suggested to be involved in the dysregulation of adipokine expression and in the consequential development of obesity-related insulin resistance and chronic inflammation [4], [5]. This hypothesis was recently confirmed when evidence was provided that hypoxia occurs in the adipose tissue of different obese mouse models and that hypoxia contributes to the endocrine dysregulations [6]–[8]. Specifically, transgenic expression of a constitutively active form of hypoxia-inducible factor 1α (HIF1A) induced inflammation in the adipose tissue of mice, which further led to an impaired glucose tolerance [9]. In human subjects reduced adipose tissue oxygen levels were found to strongly correlate with percent body fat [10].

Adipose tissue has been shown to be poorly vascularised per se [5]. In obesity, adipose tissue undergoes expansion and therefore growth and remodelling of its capillary network is required [11]. Angiogenesis is important to counteract hypoxia within expanding adipose tissue when clusters become distant form vasculature [11]. Under low oxygen levels the cells respond with an alteration in gene expression of genes which encode for proteins involved in angiogenesis, cell proliferation, apoptosis, and glucose and energy metabolism [12]. Some adipokines like angiopoietin-like 4 (ANGPTL4) and leptin have been implicated in angiogenesis [13]–[15]. Furthermore, expression of the hypoxia-sensitive transcription factor HIF1, which mediates angiogenesis and apoptosis control [16], [17], is higher in adipose tissue of obese than in lean individuals [18] and a reduced expression is associated with type II diabetes [19], [20].

Thus, the impact of hypoxia on gene expression in adipocytes seems to be central in the inflammatory response observed in obesity. However, the overall extent of transcriptional changes is unclear. In the current study whole-genome expression microarrays were used to investigate the regulation of gene expression by hypoxia in differentiated Simpson-Golabi-Behmel syndrome (SGBS) cells. As human adipose tissue material with potential to differentiate is limited, SGBS adipocytes are an excellent model to study adipocyte biology *in vitro*. Derived from the stromal cells fraction of subcutaneous adipose tissue of a patient suffering from Simpson-Golabi-Behmel syndrome, these human preadipocytes feature a long lasting and high capacity for adipose differentiation and at the same time display a gene expression pattern similar to mature fat cells [21], [22]. Although SGBS cells are a valuable and well established tool for gene expression in human fat cells [23]–[28], gene expression profiling of cultured SGBS adipocytes under hypoxic conditions has not been investigated yet.

## Materials and Methods

### Cell culture and reagents

Human SGBS preadipocytes were cultivated as described previously [21]. Briefly, cells were maintained in DMEM/Ham's F12 (1∶1) medium (Invitrogen, Paisley, UK) containing 10% fetal calf serum (FCS; Invitrogen), 100 U/ml penicillin (Invitrogen), 100 µg/ml streptomycin (Invitrogen), 33 µM biotin and 17 µM pantothenate. To differentiate SGBS cells into adipocytes, near confluent cells were washed three times with PBS and cultured in FCS-free differentiation medium: DMEM/Ham's F12 (1∶1) medium supplemented with 100 U/ml penicillin, 100 µg/ml streptomycin, 33 µM biotin, 17 µM pantothenate, 10 µg/ml human transferrin, 10 nM insulin, 100 nM hydrocortisone, 0.2 nM triiodothyronine, 25 nM dexamethasone, 500 µM 3-isobutyl-1-methylxanthine (IBMX) and 2 µM rosiglitazone (Cayman Chemical, Ann Arbor, MI, USA). After 4 days, this medium was replaced with differentiation medium excluding dexamethasone, IBMX and rosiglitazone, which was changed every 3–4 days. At day 15 after induction of differentiation, fully differentiated SGBS cells were exposed to hypoxia. To create a hypoxic environment (1% O_2_), cells were placed in a MIC-101 modular incubator chamber (Billups-Rothenberg, Inc., Del Mar, CA, USA), flushed with a mixture of 1% O_2_, 5% CO_2_ and 94% N_2_, sealed and incubated at 37°C. Adipocytes were cultured in the hypoxic environment for 3, 6 and 16 hours and the control group was cultured under normoxic conditions (21% O_2_). Cells were harvested after treatment for different time periods. Reagents were obtained from Sigma-Aldrich (St. Louis, MO, USA) unless specified otherwise.

### Microarray analysis

Total RNA was prepared from control and treated SGBS cells, in triplicate experiments, with the ‘RNeasy Lipid Tissue’ kit according to the manufacturer's instructions (Qiagen, Hilden, Germany), followed by purification of the RNA with the ‘RNeasy Mini’ kit (Qiagen). RNA quantity and purity was determined by optical density measurements (OD260/280) and RNA integrity by using the 2100 Bioanalyzer (Agilent Technologies, Palo Alto, CA, USA). Only high quality RNA was further processed. Gene expression profiling analysis was performed by the Expression Profiling Unit at the Medical University Innsbruck according to the following protocol: 500 ng total RNA was processed to generate a biotinylated hybridization target using ‘One Cycle cDNA Synthesis’ kit and the ‘One Cycle Target Labelling’ kit (Affymetrix, Santa Clara, CA, USA). All procedures were performed according to the manufacturer's protocols. In brief, total RNA was reverse-transcribed into cDNA using an anchored oligo-dTT7-Primer, converted into double-stranded cDNA and purified with the ‘Affymetrix Sample Clean-up’ kit according to the manufacturer's protocol. Thereafter, cRNA was generated by T7 polymerase-mediated *in vitro* transcription including a modified nucleotide for subsequent biotinylation. Following RNA purification, 20 µg of cRNA were fragmented at 95°C using the Affymetrix fragmentation buffer, mixed with hybridization buffer containing hybridization controls and hybridized to the ‘Human Genome U133 2.0’ arrays (Affymetrix). The arrays were stained and washed in an Affymetrix fluidic station 450. Fluorescence signals were recorded by an Affymetrix scanner 3000 and image analysis was performed with the GCOS software.

### Real-time PCR analyses

RNA (1 µg) was reverse transcribed using the SuperScript III First-Strand Synthesis Kit (Invitrogen). Expression levels of genes, which were consistently differentially expressed in response to all applied time periods of hypoxia, were assessed by real-time PCR using the SYBR® Green PCR Master Mix (Applied Biosystems, Foster City, CA, USA) and fluorescence was detected with the LightCycler® 480 System (Roche Diagnostics GmbH, Mannheim, Germany).

The primers were designed using Gene Runner software (Hastings Software, Inc.; version 3.05) and synthesized by Microsynth (Balgach, Switzerland); following sequences for the primers were used: ADM: fwd 5′-TGATGTACCTGGGTTCGCTC-3′ and rev 5′-CATCCGCAGTTCCCTCTTC-3′, ANKRD37: fwd 5′-AGAAGCTCCACTACACAAGGCA-3′ and rev 5′-AAATCCACATGACCAAGCGAG-3′, DDIT4: fwd 5′-TTGTCTTAGCAGTTCTCGCTGA-3′ and rev 5′-AAAGGCTAGGCATGGTGAGG-3′, KDM3A: fwd 5′-ATGCTGCAAAGGACACGG-3′ and rev 5′-GAACTCCATACTCTTGATGAAGACG-3′, PFKFB4: fwd 5′-GGAAATGACCTACGAGGAAATTC-3′ and rev 5′-CCAGCACATTCTCTTGCCTC-3′, PPP1R3C: fwd 5′-CCGCCTCTCTGCCTAATGA-3′ and rev 5′-CCCAGGAAACTCTTCACAGGT-3′, VEGFA: fwd 5′-GCGCAAGAAATCCCGGTATA-3′ and rev 5′-CGCGAGTCTGTGTTTTTGCA-3′, WDR73: fwd 5′-GAGTCCTTGAATGGATTGATGAC-3′ and rev 5′-TGAAATCTCTTTCTGGGAATAAGC-3′, ZNF395: fwd 5′-CCTTTCCTGCTGGACGAA-3′ and rev 5′-TTTGATGCCCACAATGGA-3′, and TBP: fwd 5′-GGGAGCTGTGATGTGAAGTTT-3′ and rev 5′-AAGGAGAACAATTCTGGGTTTG-3′. A thermal profile of an initial 10 min melting step at 95°C, followed by 40 cycles of 15 s at 95°C and 1 min at 60°C was used. A melting curve profile was processed after each run to confirm specific transcripts. All reactions were performed in triplicates and the samples were normalized to the endogenous reference TBP values. The results are expressed as fold changes of cycle threshold value relative to controls using the 2^-ΔΔCt^ method.

### Statistical analyses

Microarray data analysis was performed in R (http://www.r-project.org) using packages from the Bioconductor project [29]. Functions from the affyPLM package were used for quality assessments of the microarrays and only good quality arrays were further analyzed. GeneChip raw expression values were normalized and summarized using the GC robust multi-array average (GCRMA) method [30]. Enrichment analysis for the Gene Ontology (GO) categories were computed via the gene ontology tree machine (www.geneontology.org) and the multiple test adjustment proposed by Benjamini and Hochberg [31]. The adjusted p value indicates the significance of enrichment. Further in silico analyses were conducted using the gene ontology software package ExPlain 3.0™ with the integrated Match™ tool and the linked TRANSFACPro® and BioKnowledge Library (BKL) as well as predefined gene sets and position weight matrix (PWM) profiles provided by BIOBASE (www.biobase-international.com). To identify binding sites in a query set of differentially regulated genes (Yes-set), we used a background gene set (No-set) and a predefined PWM profile consisting of 656 PWMs for human transcription factor binding sites. Matched matrices with a Yes/No ratio >1 were considered overrepresented in the query set. Real-time PCR data analyses were performed using the statistical software package SPSS 11.0 for Windows, SPSS Inc. Chicago, IL, USA. The results are expressed as mean values ± SEM. Statistical differences between groups were analyzed using unpaired two-tailed Student's *t* tests.

## Results

Microarray analysis showed 10 significantly differentially regulated mRNAs after 3 hours, 52 after 6 hours and 514 after 16 hours incubation of the cells under low oxygen levels compared to the untreated control, which met a fold-change of >2 and a p value of <0.05.

To gain insight into the biological processes and molecular functions of the hypoxia-regulated genes we considered the 514 genes regulated after 16 hours of hypoxia for statistical analysis with the gene ontology tree machine to classify the regulated genes according to their function. The analyzed genes are involved in several biological processes, and significance of enrichment was evaluated for glycolysis (10 genes), response to hypoxia (16 genes), regulation of cellular component movement (17 genes), response to nutrient levels (16 genes), regulation of cell migration (13 genes) and transcription regulator activity (60 genes). Twenty-four out of the 514 regulated genes distributed by gene ontology (GO) classification are involved in more than one pathway (table 1).

**Table 1:** Significantly regulated genes classified according to their function in SGBS under hypoxia for 16 hours (>2-fold).

Biological process	p-value	Gene symbol
Glycolysis	0.0006	INSR, ENO2, PFKL, PGK1, ALDOC, HK2, GPI, HK1, PFKP, TPI1
Response to hypoxia	0.0006	EPAS1, VLDLR, PLOD1, VEGFA, HMOX1, ANGPTL4, TFRC, ADM, DDIT4, PLOD2, ALDOC, FLT1, BNIP3, MT3, EGLN3, EGLN1
Regulation of cellular component movement	0.0038	ABHD2, NF2, MAP2K1, SPHK1, VEGFA, BCAR1, HMOX1, ETS1, KISS1R, INSR, SP100, FLT1, ARAP1, BCL6, TAC1, SCAI, SCARB1
Response to nutrient levels	0.0073	RARA, VLDLR, AQP3, LIPG, HMOX1, TFRC, ADM, JUN, PTGS2, INSR, SUOX, C14orf104, STC2, LEP, CDKN2D, STC1
Regulation of cell migration	0.0302	ABHD2, INSR, NF2, FLT1, MAP2K1, SPHK1, SCARB1, TAC1, SCAI, VEGFA, BCAR1, HMOX1, KISS1R
Transcription regulator activity	0.0164	HOXA7, ZNF35, ZNF207, HIVEP3, MXI1, EPAS1, UBP1, CDKN1C, CNOT8, PPARGC1B, KHDRBS1, ETV5, PBX1, ATF7IP, MEF2A, CEBPA, FOSL2, ETS1, RORA, JUN, E2F7, CAMTA2, HOXA4, SP100, ZNF167, BCL6, RFX5, CREB1, MEIS1, SNAPC5, CEBPD, SAP30, TGIF1, RARA, TMF1, TCEB3, RFX2, NFIA, WDR77, ATF3, CBL, BCL10, BTG1, ID3, TRIB3, SERTAD2, SMARCC1, HOXA9, NFIL3, NAB2, DDX5, SCAI, TFDP2, SMAD1, MAFF, RFXAP, FOXQ1, HEY1, KLF7, FUBP1

Gene ontology (GO) tree machine with multiple test adjustment proposed by Benjamini & Hochberg (1995) was used for the enrichment analysis. The adjusted p value indicates the significance of enrichment.

**Figure 1:**
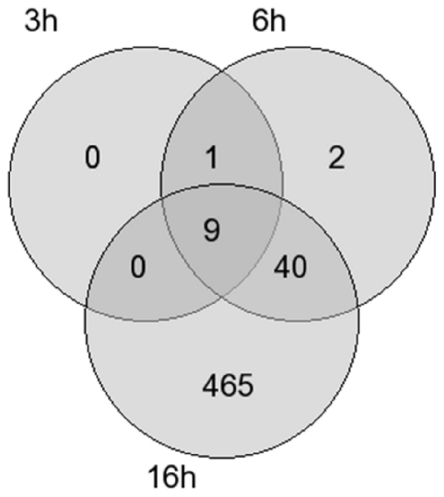
Venn diagram of the hypoxia-regulated genes in human adipocytes. Microarray analysis resulted in 10 differentially regulated genes after 3 hours, 52 differentially regulated genes after 6 hours and 514 differentially regulated genes after 16 hours cultivation of the cells in a hypoxic environment (1% O_2_). In the intersection of the circles the number of genes commonly regulated to the corresponding time points is indicated. The genes included in this analysis showed at least a 2-fold change in the expression compared to the control with a p value <0.05.

**Figure 2:**
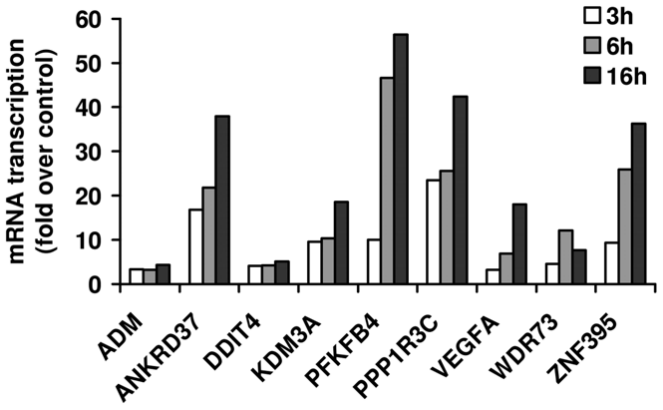
Transcriptional induction in response of hypoxia. Transcriptional induction under hypoxic condition (1% O_2_) is shown for the consistently differentially expressed genes after 3, 6 and 16 hours cultivation of the mature SGBS adipocytes. The fold change values are shown relative for the expression levels under normoxic conditions. Only genes, which met a p value of <0.05 compared to the untreated control, are shown.

**Figure 3:**
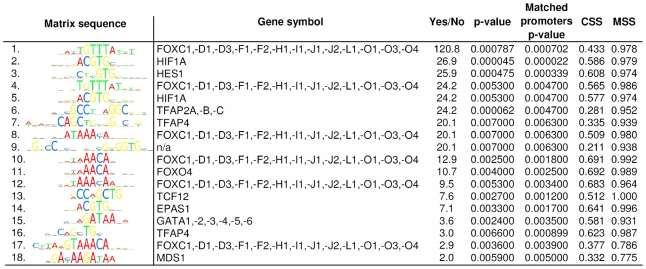
Transcription factors binding sites identified within the promoter regions. The figure displays the sequences of matched position weight matrices (PWMs) together with the corresponding gene symbols of the transcription factors, which are overrepresented in the promoters of the 9 upregulated genes. The PWMs are ranked according to their “Yes/No” ratio, which is defined as the ratio of the average number of putative binding sites per 1000 bp, given for the query (Yes) and the background (No) sets. Overrepresentation is defined as a Yes/No ratio greater than one. Significance of the representation value is measured by the p-value derived from a binomial distribution. Matched promoters p-value assesses the statistical significance of the number of promoters in the query set that have at least one predicted site compared to that of promoters in the background set. Matrix similarity score (mss) and core similarity score (css) are indicated for comparison.

Further analysis of the results obtained from the microarrays showed that 9 mRNAs were consistently differentially expressed at 3, 6 and 16 hours of 1% O_2_ (figure 1). These genes encode for adreno­medullin (ADM), ankyrin repeat domain 37 (ANKRD37), DNA-damage-inducible transcript 4 (DDIT4), lysine(K)-specific demethylase 3A (KDM3A), 6-phosphofructo-2-kinase/fructose-2,6-bip­hosphatase 4 (PFKFB4), protein phosphatase 1 regulatory (inhibitor) subunit 3C (PPP1R3C), vascular endothelial growth factor A (VEGFA), WD-repeat domain 73 (WDR73), and zink finger protein 395 (ZNF395). The transcriptional induction values of these genes obtained from the microarray data are shown in figure 2. Thereupon we analyzed proximal promoter sequences (-700 bp to +300 bp with respect to the transcription start site) of the corresponding genes, looking for transcription factor binding sites. Applying the F-Match tool integrated in the ExPlain software we used a predefined set of 561 human housekeeping genes, from which the set of 514 upregulated genes after 16h were excluded, as background and the PWM profile mentioned above for the calculation. The result was filtered according to their p-values (<0.007) and Yes/No ratio (>1.3) and ranked according to their Yes/No ratio which represents their abundance in the set of 9 genes (ADM, ANKRD37, DDIT4, KDM3A, PFKFB4, PPP1R3C, VEGFA, WDR73 and ZNF395) compared to the background. The 18 best hits among the matched PWMs and the graphical output of the corresponding consensus sequences are displayed in figure 3. At position two, five and fourteen, according to the ranking, we found PWMs representing hypoxia response.

**Figure 4:**
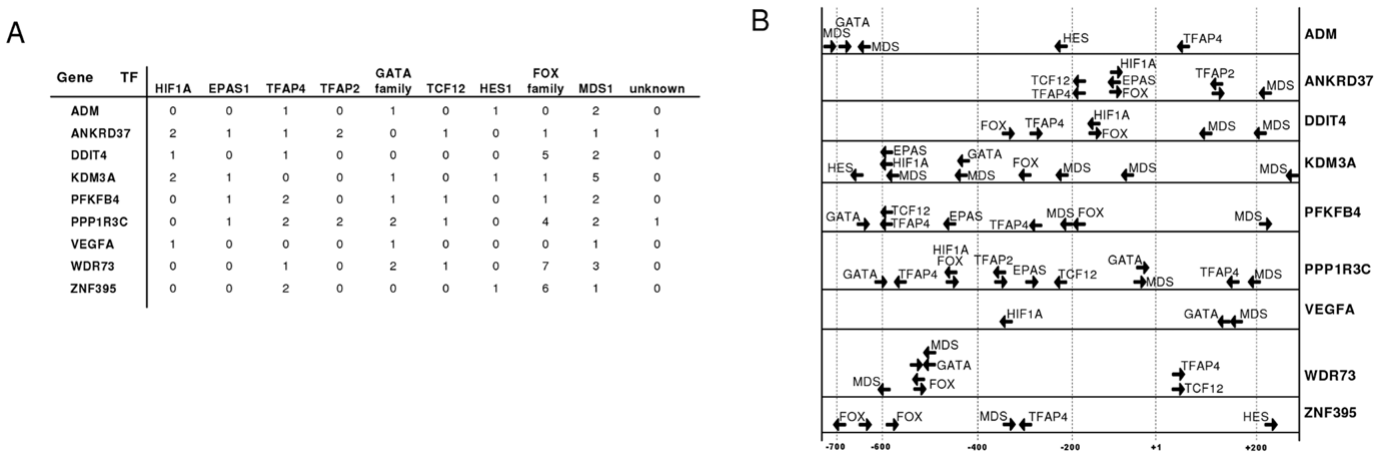
Binding sites for transcription factors within promoter regions. (A) Tabulated view of all matched matrix sequences for transcription factors (TF) as displayed in figure 3. Hits for transcription factors which were generated according to different matrices representing the same transcription factor were summed up. (B) Schematic representation of matched binding sites for transcription factors (arrows) within proximal promoters of the eight verified hypoxia-induced genes and WDR73. Overlapping binding sites of different matrices which represent the same transcription factor (family) are indicated as single site in the figure. Binding sites of the unknown factor were omitted.

We identified at least one binding site for the human hypoxia-inducible factor 1 alpha subunit (HIF1A) and the endothelial PAS domain protein 1 (EPAS1), also known as HIF2A, a transcriptional activator that acts in cell proliferation, cell migration, hemopoiesis and angiogenesis [32]–[34], sharing the consensus sequence 5′-ACGTG-3′ in ANKRD37, DDIT4, KDM3A, PPP1R3C, PFKFB4 and VEGFA (figure 4 A). Interestingly, several PWMs for the forkhead transcription factor superfamily and one specific for forkhead box O4 (FOXO4), which mediates HIF1A and VEGF downregulation [35] were also recognized. In case of ANKRD37, DDIT4 and PPP1R3C binding sites of FOX- and HIF-family transcription factors overlap (figure 4 B). Apart from that, binding sites for TCF12 and the repressors HES1 and MDS1 are located within the mentioned promoters. We also identified PWMs for transcription factor activating enhancer binding protein 2 (TFAP2A), which is known to activate ADM expression in monocytic leukemia cells [36], for TFAP4 and for transcriptional activators of the GATA binding protein family (figure 3 and 4).

Moreover, reinvestigating the subset of 10 genes upregulated after 3 h and 6 h of hypoxia (figure 1), BHLHE41, the only gene, which was excluded by the Venn-Diagramm according to a lack of upregulation after 16 h, which codes for the Class E basic helix-loop-helix protein 41 (BHLHE41) mediating sleep length and apoptosis [37], [38], is also known to be activated by HIF-1 [39]. A functional classification of these ten genes by conducting GO annotation with the manual curated BKL database supplied by BIOBASE significantly assigned six of these ten genes (ADM, DDIT4, KDM3A, PFKFB4, VEGFA and BHLHE41) to response of hypoxia and oxygen levels with p-values of 1.9 e-8 and 2.0 e-8 respectively and seven of ten to response to stress and chemical stimulus (table 2).

**Table 2:** Functional classification of 10 genes upregulated after 3 h of hypoxia by GO annotation.

Biological process/GO ID	p-value	Gene symbol
Response to hypoxia/GO: 0001666	3.83E-10	ADM, BHLHE41, DDIT4, KDM3A, PFKFB4, VEGFA
Response to oxygen levels/GO:0070482	4.20E-10	ADM, BHLHE41, DDIT4, KDM3A, PFKFB4, VEGFA
Response to stress/GO:0006950	4.21E-05	ADM, BHLHE41, DDIT4, KDM3A, PFKFB4, VEGFA, ZNF395
Response to chemical stimulus/GO:0042221	1.01E-04	ADM, BHLHE41, DDIT4, KDM3A, PFKFB4, VEGFA, ZNF395

The 10 genes upregulated after 3 h of hypoxia were classified according to their biological processes and gene ontology (GO) IDs (www.geneontology.org) by GO annotation, using manual curated GO groups provided by the Biobase software package Explain.

**Figure 5:**
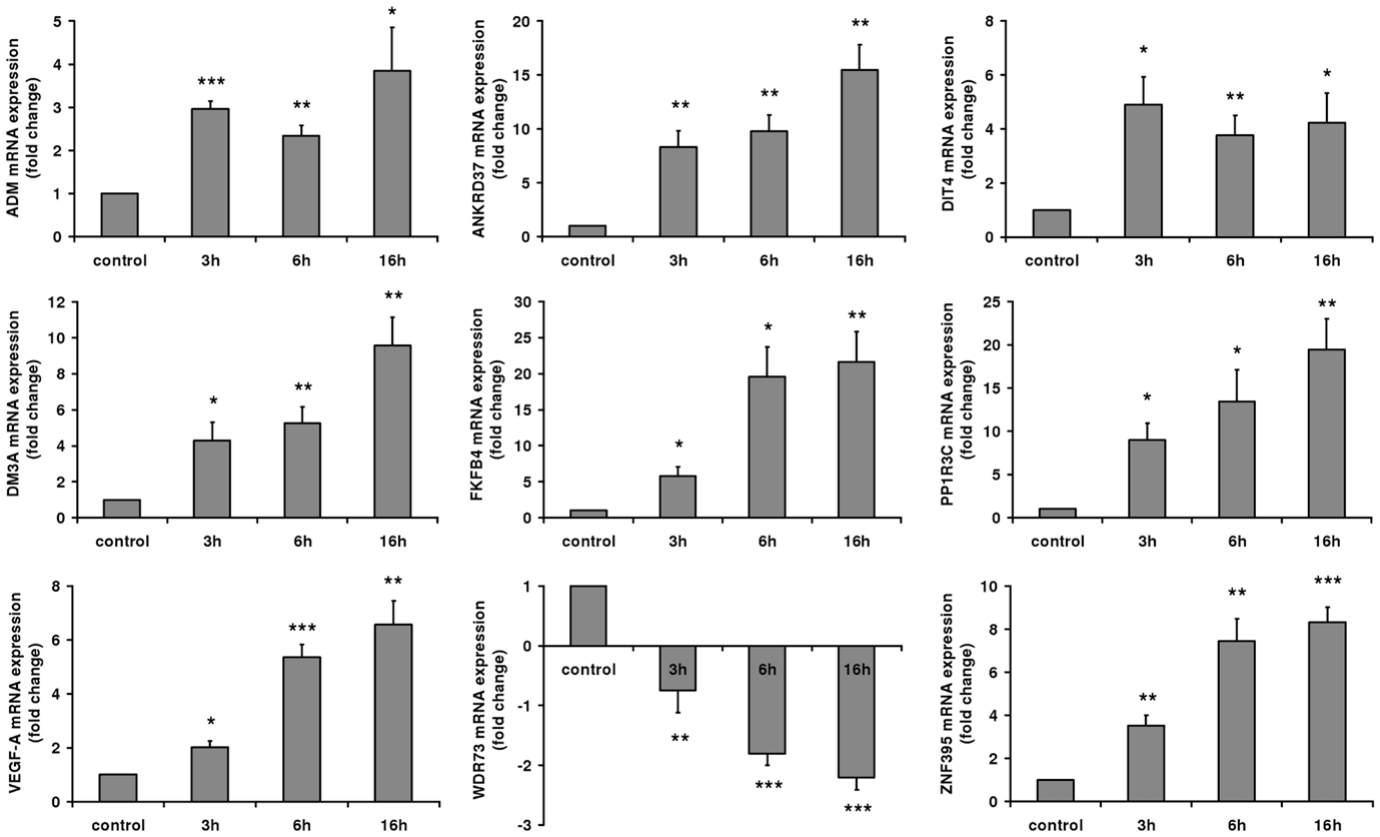
Transcriptional induction according to real-time PCR analysis. Real-time PCR analysis was performed analysing mRNA levels of the 9 genes differentially regulated after 3, 6 and 16 hours treatment under hypoxic conditions (1% O2). Results of 4–6 independent experiments each performed in triplicate are expressed as mean values ± SE. Values are depicted relative to the untreated control. *** p<0.001; ** p<0.01; * p<0.05.

To prove the validity of the microarray data, the set of the 9 significantly regulated genes after all investigated time periods (3, 6, 16 h) under hypoxia was also determined by real-time PCR. These validation experiments were equally performed for all time periods used in the microarray analysis and were assessed by repeated experiments (figure 5). Overall, the regulation patterns measured in the real-time PCR experiments reflected the results of the microarray data, with one exception: In the case of WDR73 of the validation experiments a significant regulation of gene expression in the opposite direction was observed compared to the microarray data. Given the verified transcriptional upregulation of ADM, ANKRD37, DDIT4, KDM3A, PFKFB4, PPP1R3C, VEGFA and ZNF395 on the one hand and their clustering in response to hypoxia according to gene ontology, we were interested in a potentially coordinated expression. Reinvestigating the 514 genes upregulated after 16 h, we noticed that HIF2A is on the one hand a transcription factor, binding to upregulated genes as diplayed in figure 3 and 4, and, on the other hand, upregulated itself in SBGS adipocytes after 16h of hypoxia. According to our binding site analysis, HIF1, HIF2A and TFAP4 together bind all eight genes which were verified by real time PCR to be upregulated under hypoxia (figure 4) and therefore might be sufficient to induce their activation.

## Discussion

In the present study we identified several hypoxia-regulated genes, shown for the first time in human adipocytes, by using whole-genome microarrays. The genes discovered are in part targets of the hypoxia-sensitive transcription factors HIF1 and HIF2A and are involved in crucial pathways within the cell and may contribute to the initiation of mechanisms, which further promote the development of obesity-related diseases such as type 2 diabetes and the metabolic syndrome.

Our study showed that hypoxia for 16 hours leads to the significant regulation of over 500 transcripts in human adipocytes and activates several pathways and biological processes involved in the supply of energy. The up-regulation of genes involved in glycolysis to obtain an adequate energy level is used as a survival strategy for the cell [40]. Furthermore, our data demonstrate that genes involved in the regulation of cellular component movement and cell migration are regulated under hypoxic conditions. Further, a significant enrichment of regulated genes involved in transcription regulator activity and response to hypoxia was observed. The regulation of genes involved in these processes is suggested to lead to adaptive changes, which allow the cell to gain more energy and lead to the delivery of oxygen [12]. Interestingly, we could not identify an accumulation of hypoxia-regulated genes involved in inflammation, although hypoxia in adipose tissue is suggested to lead to a chronic inflammatory state [41]. Of note, white adipose tissue consists of different cell types, such as pre-adipocytes, macrophages, endothelial cells, fibroblasts and leukocytes, which all contribute to the production and release of cytokines [42], [43]. It is also suggested, that the response to hypoxia differs depending on the cell type and the tissue environment [44], [45]. Therefore, the contribution of adipocytes to the total amount of secreted factors from adipose tissue *in vivo* which lead to a chronic inflammation is not fully understood.

From the large number of hypoxia-regulated transcripts, nine genes were found to be differentially expressed after all time periods of hypoxia (3, 6 and 16 hours) investigated in our microarray study. Our findings in SGBS adipocytes are consistent with previous reports observed in other cell types. A hypoxia-induced regulation was described for ADM [46]–[50], ANKRD37 [51], [52], DDIT4 [51], [53], [54], KDM3A [51], [55]–[57], PFKFB4 [58], PPP1R3C [59], and ZNF395 [60]. ADM, ANKRD37, DDIT4, KDM3A, PFKFB4, PPP1R3C and VEGF have also been described to be direct HIF-targets [50], [51], [53]–[56], [58], [59], [61], [62]. However, only the hypoxia-induced regulation of VEGF has been described for adipocytes so far [63], [64]. So our results enlarge this picture as we could demonstrate for the first time that hypoxia induces also the gene expression of ADM, ANKRD37, DDIT4, KDM3A, PFKFB4, PPP1R3C and ZNF395 in SGBS adipocytes. As the SGBS cells, used for this study have been proven in several human adipocyte biology studies to have characteristics very similar to adipocytes [21]–[28] we consider our findings also relevant for adipocytes in general.

VEGF is essential for the development of the vascular system and promotes angiogenesis [65] and also ADM has been shown to induce angiogenesis [66]. Both, the growth of adipose tissue and hypoxic conditions require the development of the vascular network. Concentrations of serum VEGF have been described to positively correlate with body mass index and body weight reduction led to a decrease in VEGF circulating levels [67]. This angiogenic factor has also been suggested to be involved in the development of the comorbidities associated with obesity [68], [69]. Furthermore, plasma concentration of the antiadipogenic factor ADM increases with obesity, the incidence of type 2 diabetes, cardiovascular diseases and inflammation [70], [71]. The expression of ADM and DDIT4 in adipocytes, which is induced in response to different situations of cellular stress is in addition to hypoxia also regulated by insulin [72], [73] and in terms of DDIT4, whose overexpression may participate to the development of insulin resistance, depends on the transcription factor HIF-1 [73].

Further, also KDM3A has been described as a regulator of genes involved in energy expenditure and fat storage [74], [75]. Therefore, these identified genes may play a crucial role in the regulation of obesity and the metabolic syndrome.

The remaining hypoxia-regulated genes that were identified are involved in different cellular functions: protein-protein interaction and signalling (ANKRD37) [76], glycolysis (PFKFB4) [77], glucogen accumulation which is suggested as metabolic longterm adaption to hypoxia (PPP1R3C) [59], and regulation of transcription (ZNF395) [78], [79]. The function of WD-repeat proteins differs from signal transduction, regulation of transcription, and apoptosis, but the function of WDR73 still remains unknown [80], [81]. However, only hypoxia-induced regulation of WDR73 could not be confirmed by real-time PCR. Compared to fold change expression of other genes, a small but significant regulation of WDR73 gene expression in the opposite direction was observed by real-time PCR compared to the microarray data. This may be due to different oligonucleotides used for PCR, which are specific for exon 2 and 4, and those for microarray analysis, detecting alternative splicing products. However nothing is known about possible alternative splicing mechanisms of WDR73 RNA. Interestingly, we did not detect a binding site for hypoxia-inducible factors 1 and 2 according to our cut off settings. Both findings argue against a role of WDR73 during hypoxia. However we do not rule out that there are further hypoxia responsible elements (HREs) for those factors, as we also did not detect the HREs described by Garayoa et al. in ADM [50] according to our settings. Therefore future studies may clarify the regulation and function of WDR73.

Furthermore we could not only show that most genes, upregulated by hypoxia, harbour binding sites for HIF1A and EPAS1, but also that EPAS1 is upregulated after 16h of hypoxia, indicating a positive feedback mechanism. Looking for general composite promoter models within these genes we did not detect a striking conformity. However, we revealed binding sites for FOXO4 and the forkhead box superfamily within almost every proximal promoter of the upregulated genes. FOXO transcription factors mediate anti-angiogenic effects [82] and play a central role during oxidative stress [83]. In addition, the transcription regulator FOXO4 is known to downregulate the expression of EPO, VEGFA and HIF1A [35]. In this context, the direct overlap of FOX- and HIF-binding sequences within ANKRD37, DDIT4 and PPP1R3C may suggest an interaction or a competition of these factors for binding to the respective promoter elements. We suppose that the FOX-family of transcription factors may play an important role for gene regulation during hypoxia in SGBS adipocytes, likely by impeding excessive expression after hypoxic treatment. However, further studies regarding protein expression patterns in adipocytes will be necessary to test this hypothesis.

Taken together, we demonstrated that hypoxia has an extensive effect on gene expression of SGBS adipocytes, which responded with the regulation of genes involved in different crucial biological processes. In addition, the identified hypoxia-regulated genes have been in part suggested to be involved in the regulation of obesity, the incidence of type 2 diabetes, and the metabolic syndrome.

However, adipose tissue *in vivo* consists also of other cell types than the investigated adipocytes. Therefore, additional studies using primary cells e.g. derived from lipoaspirates are needed to be performed to elucidate the relation of whole adipose tissue hypoxia and the chronic inflammation observed in obesity. Nevertheless, we have now established a comprehensive gene expression profile for these adipocytes under hypoxia.
